# Calmodulin assists during co‐translational folding of the K_V_7.2 channel calcium responsive domain

**DOI:** 10.1002/pro.70552

**Published:** 2026-04-08

**Authors:** Arantza Muguruza‐Montero, Jack R. Tait, Sara M‐Alicante, Ane Metola, Eider Nuñez, Janire Urrutia, Vanda Sunderlíková, Alexandros Katranidis, Gunnar von Heijne, Sander J. Tans, Alvaro Villarroel

**Affiliations:** ^1^ Instituto Biofisika, CSIC‐UPV/EHU Leioa Spain; ^2^ Department of Physiology, Faculty of Medicine and Nursery University of the Basque Country (UPV/EHU) Leioa Spain; ^3^ AMOLF Institute, Science Park 104 Amsterdam Netherlands; ^4^ Department of Biochemistry and Biophysics Stockholm University Stockholm Sweden; ^5^ ER‐C‐3 Structural Biology, Forschungszentrum Jülich Jülich Germany; ^6^ Science for Life Laboratory Stockholm University Solna Sweden; ^7^ Bionanoscience Department Kavli Institute of Nanoscience Delft, Delft University of Technology Delft Netherlands

**Keywords:** calmodulin, co‐translational folding, folding, K_V_7.2

## Abstract

*In vivo*, the majority of nascent protein chains begin folding during translation in order to reach their native structure. While the importance of co‐translational folding has become increasingly clear, the specific mechanisms underlying the coordination between the ribosome, the nascent chain and interacting partners are still uncertain. Here, we show that calmodulin (CaM) plays a prominent role at discrete steps of the co‐translational folding pathway of the calcium responsive domain (CRD) of the human neuronal K_V_7.2 ion channel, providing grounds for the proposal of a likely folding pathway. By combining force profile analysis and single‐molecule force spectroscopy techniques, we found that CaM, in a calcium‐dependent manner, affects early folding events involving three key α‐helices in the CRD. In addition, this study suggests that CaM at early stages participates in the formation of metastable helical hairpins, as part of the co‐translational folding pathway. These findings expand on the role of CaM as a key regulator of co‐translational folding.

## 1 INTRODUCTION

Calmodulin (CaM) is a small, highly flexible protein composed of two globular lobes (N‐ and C‐lobe) connected by a central linker. Acting as the primary calcium (Ca^2+^) sensor in eukaryotic cells, CaM translates fluctuations in intracellular Ca^2+^ concentration into conformational changes that regulate a wide spectrum of target proteins unable to bind calcium directly (Alaimo & Villarroel, 2018). Ubiquitously expressed across cellular compartments, CaM governs numerous physiological processes through its ability to recognize and stabilize diverse structural motifs. Beyond its canonical signaling functions, CaM also exhibits chaperone‐like activities: it prevents aggregation of K_V_7.1–K_V_7.2 channel fragments expressed in bacteria (Bernardo‐Seisdedos et al., 2018; Wiener et al., 2008), participates in subunit assembly and protein translocation within the nucleus and endoplasmic reticulum (Bernardo‐Seisdedos et al., 2018; Etzioni et al., 2011; Ghosh et al., 2006; Gold et al., 2013; Shamgar et al., 2006; Shao & Hegde, 2011), and maintains the translocation competence of small cytosolic precursors by limiting non‐specific interactions with other polypeptide‐binding proteins (Shao & Hegde, 2011). CaM association with K_V_7 channels is particularly crucial for proper trafficking to the plasma membrane (Alaimo et al., 2009; Cavaretta et al., 2014; Etxeberria et al., 2008; Ghosh et al., 2006; Liu & Devaux, 2014; Shamgar et al., 2006). Mutations within the calcium‐responsive domain (CRD) of K_V_7.2 that disrupt CaM binding cause endoplasmic reticulum retention and reduced ionic currents, underlying forms of benign familial neonatal epilepsy (BFNE) and severe epileptic encephalopathy (Etxeberria et al., 2008; Tran et al., 2020). These findings suggest that CaM may assist the co‐translational folding of the CRD; however, the precise molecular mechanisms through which CaM exerts this influence remain largely unresolved (Urrutia et al., 2021).

The calcium‐responsive domain (CRD) resides within the extended cytosolic C‐terminal region of the K_V_7.2 channel and comprises three α‐helices: hA, hTW, and hB. When complexed with CaM, the CRD adopts an antiparallel hairpin conformation, in which the C‐ and N‐lobes of CaM engage helices hA and hB, respectively (Bernardo‐Seisdedos et al., 2018). The relative mobility between hA and hB is essential for Ca^2+^‐dependent gating and signal transduction (Bernardo‐Seisdedos et al., 2018; Nuñez et al., 2023). In contrast, the structural and functional contributions of the intervening hTW helix remain less well defined. Nuclear magnetic resonance (NMR) analyses indicate that hTW is highly dynamic (Bernardo‐Seisdedos et al., 2018), and currently available cryo‐EM and X‐ray structures of K_V_7 channels (PDBs 6FEG, 6FEH, 7CR3, 7CR4, 7CR7, 8J01, 8J02, 8J04, 8J05, 8W4U) reveal no direct engagement of this helix within the canonical CaM‐binding clefts (Bernardo‐Seisdedos et al., 2018; Li et al., 2021; Ma et al., 2023). Functional evidence suggests that hTW plays a conditional stabilizing role: while dispensable under normal conditions, it becomes crucial when CaM interaction at either hA or hB is compromised, implying a compensatory mechanism that safeguards channel function (Gomis‐Perez et al., 2015).

The CRD, devoid of transmembrane segments, constitutes an autonomously folding domain capable of attaining its native conformation independently of the full‐length channel. This characteristic renders the CRD a tractable model system for dissecting the molecular determinants of folding and for elucidating the role of its interacting partners. In particular, its well‐defined interaction with CaM provides a unique opportunity to explore how this essential calcium sensor modulates the co‐translational folding and structural maturation of a complex cytosolic domain under physiologically relevant conditions.

In the present work, we employ the CRD as a model system to explore how CaM may influence co‐translational folding. Our results support a plausible folding pathway in which CaM promotes the sequential and transient formation of helical hairpins – first between hA and hTW, and later between hTW and hB‐ in a Ca^2+^‐dependent manner. These findings suggest that CaM can assist the co‐translational folding and stabilization of emerging CRD intermediates, offering a mechanistic framework that can be further tested in the context of the full‐length channel.

## 2 RESULTS

### 2.1 Impact of calmodulin on the *in vivo* force profile of K_V_7.2 CRD

**FIGURE 1:**
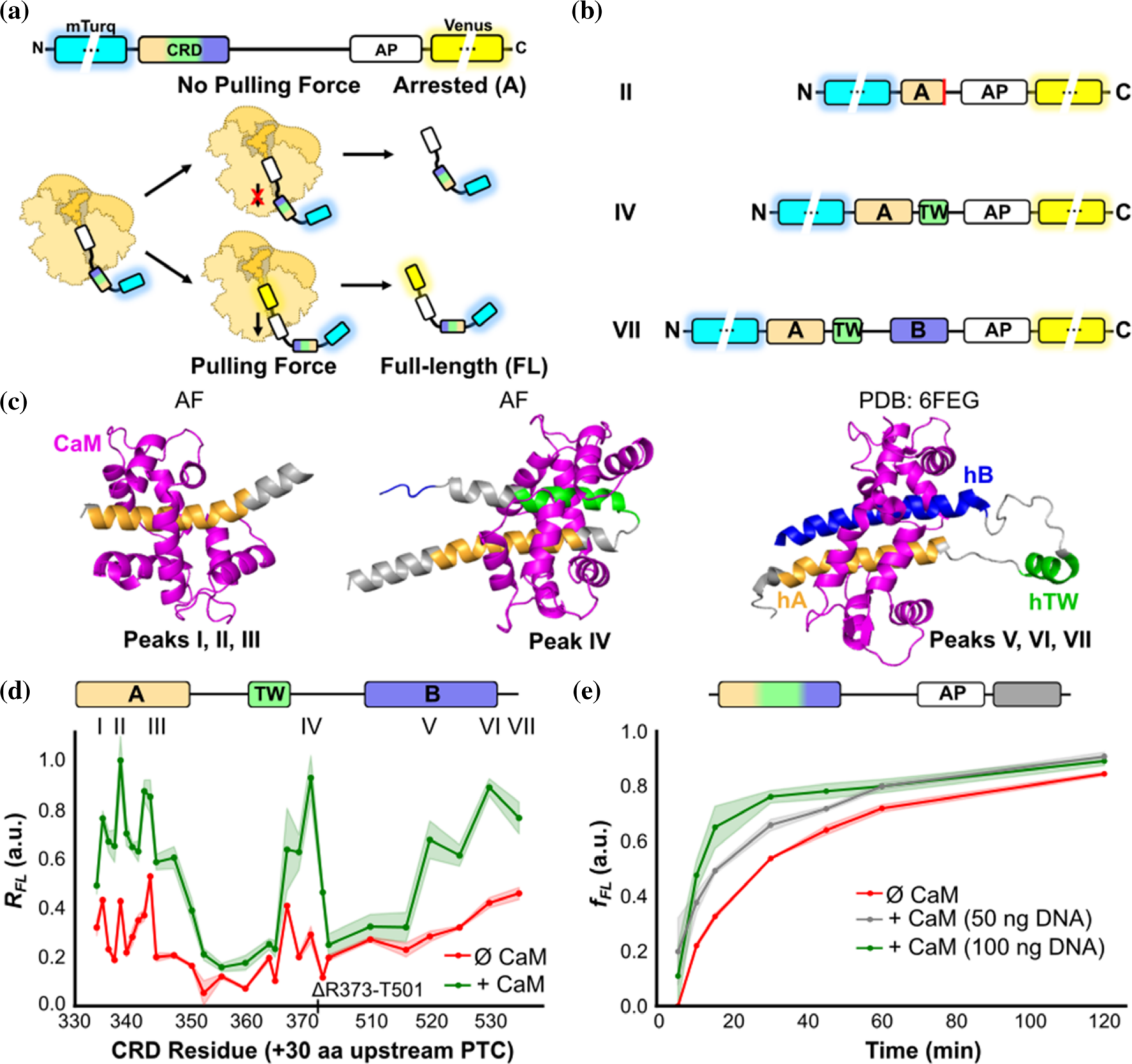
Three CaM‐dependent peaks revealed by the *in vivo* force profile, which correspond to the exit of the CRD helices (A, TW and B) from the ribosomal tunnel. (a) Schematic representation of FPA. Blue and yellow fluorescent proteins have a halo indicating its fluorescence, and are not represented in scale. CRD, calcium responsive domain; AP, arrest peptide. (b) Schematic representation of variants associated with the indicated peaks in the *in vivo* FPA. Helix A in the construct at peak II is incomplete, which is indicate by a red line. (c) Structural representation of AlphaFold (AF) predictions of hA and hA‐hTW in complex with CaM and NMR CRD/CaM structure (PDB: 6FEG). (d) *In vivo* force profiles for the CRD alone (red) and after co‐expression with CaM (green). Shaded regions represent SEM (*n* = 4–12). (e) *In vitro* time‐course pulling‐force assay of the CRD without CaM (red) and with varying amounts of CaM cDNA (50 ng in gray and 100 ng in green). Shaded regions represent SEM (*n* = 3).

To pinpoint the key translation stages where folding is promoted by CaM, we employed force profile analysis (FPA), taking advantage of the force sensitivity of translational arrest peptides (APs) to monitor co‐translational folding (Goldman et al., 2015; Ismail et al., 2012). APs are sequences that bind in the interior of the ribosomal exit tunnel, preventing chain elongation. The stalling efficiency is highly dependent on the external force applied to the nascent chain when the final residue in the AP is translated (Goldman et al., 2015; Ismail et al., 2012). Such pulling forces can be generated by the folding of the nascent chain nearby or inside the ribosomal exit tunnel (Goldman et al., 2015; Ismail et al., 2012). APs allow generating highly sensitive biosensors for reporting on co‐translational folding events: the fraction of full‐length protein (*f*_FL_) serves as a proxy for the folding propensity of the newly synthesized nascent chain at each stage of translation, as shown previously (Nilsson et al., 2015) (Figure 1a).

Here, we generated a library of 29 variants containing K_V_7.2 CRD sequences of increasing length, flanked by an N‐terminal mTurquoise2.1 fluorescent protein and a C‐terminal mcpVenus173 fluorescent protein downstream of the SecM (*Ec‐Ms*) AP (Farías‐Rico et al., 2017) (Figure 1b) (from now on referred to as Turquoise and Venus, respectively). In this experimental setup, two main products are obtained: an arrested (A) product from the protein labeled only with Turquoise and a full‐length (FL) protein that is labeled with both Turquoise and Venus (Figure S4). This enabled measurement of the relative full‐length protein (*R*_FL_) by the ratio of yellow: blue fluorescence intensity, which in turn allowed us to directly monitor the influence of CaM on folding of nascent K_V_7.2 CRD proteins during translation. AlphaFold Multimer (AF) was used to predict the structures of these 29 variants and the NMR structure (PDB: 6FEG) was taken into consideration as the native folded conformation of CRD/CaM complex (Figure 1c).

*In vivo* co‐expression of the library constructs with CaM resulted in a force profile with three clear folding transitions (marked by increases in *R*_FL_ at peaks I–III, IV and V–VII; Figure 1d, green). Considering the length of the ribosome exit tunnel (≈30 residues; Jenni, 2003; Voss et al., 2006), these transitions correspond to the sequential exit from the tunnel vestibule of each of the three α‐helices comprising the CRD. AF also predicted three main folding transitions corresponding to the appearance of each helix (Figure S6). Compared to the final folded state, which exhibits a high confidence score (ipTM + pTM = 0.79) likely due to its well‐defined structure, the folding transition models show lower confidence scores (ipTM + pTM ≈ 0.3; Table S1), consistent with the intrinsic flexibility of nascent chains and the lack of structural data. In particular, the first transition (corresponding to folding of helix hA) is split into three sub‐peaks (I, II and III). Notably, sub‐peaks II and III align with the emergence of two hydrophobic residues that are pivotal for CaM binding within the IQ motif of hA (**I** QSA **W** R, hydrophobic residues are underlined) in K_V_7.2 (Figure S1) (Muguruza‐Montero et al., 2021). Crucially, in the absence of CaM, we observed a global decrease in folding force across the entire CRD (Figure 1d, red). This implies that while the three α‐helices retain some capacity to fold independently, these transitions are significantly dependent on the presence of CaM.

To explore folding in the absence of cellular context, *in vitro* FPA was performed. Similarly to *in vivo*, *in vitro* FPA showed an *f*_FL_ increase at almost every translation stage in the presence of CaM (Figure S4). Both profiles were qualitatively similar in terms of CaM dependency and number of major peaks, although the resolution for the *in vivo* FPA was more favorable.

We addressed how CaM abundance affected the process *in vitro* (Figure 1e), using a variant which ensured the complete exit of the whole CRD (L21, see Materials and Methods; Figure S1). After 2 h, we observed accumulation of FL protein even in the absence of CaM expression (Figure 1e, red line). Significantly, the production of FL protein accelerated in a CaM‐dependent manner (Figure 1e). These results reinforce the concept of a role of CaM in assisting CRD co‐translational folding and suggest the existence of three steps, corresponding with the sequential exit of hA, hTW, and hB from the ribosomal tunnel.

### 2.2 Ca^2+^ binding to CaM impacts on co‐translational folding

The role of CaM as a crucial Ca^2+^ modulator in various physiological pathways prompted us to investigate the role of Ca^2+^ binding. For this purpose, we employed two CaM mutants unable to bind Ca^2+^ at the N‐lobe (CaM12) or the C‐lobe (CaM34), by replacing the aspartate with alanine in the first position of the EF‐hands of the N‐ or C‐lobe, respectively (Geiser et al., 1991; Keen et al., 1999).

**FIGURE 2:**
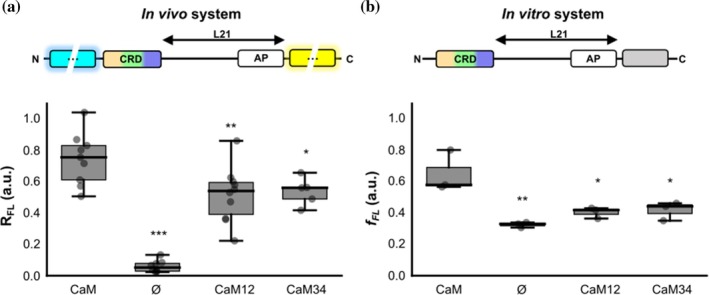
*In vivo* and *in vitro* pulling‐force assays with CaM variants unable to bind Ca^2+^ at the N‐ or C‐lobes. (a) Box plot of the *R*_FL_ for the CRD L21 folding biosensor *in vivo* without CaM and co‐expressed with CaM, CaM12 and CaM34, as indicated. Whiskers indicate SEM (*n*_CaM WT_ = 9; *n*_Ø_ = 8; *n*_CaM12_ = 10; *n*_CaM34_ = 5). (b) Box plot of the *f*_FL_ of *in vitro* assay of the CRD L21 folding biosensor alone and co‐expressed with CaM lobe mutants at 1:1 ratio. Whiskers indicate SEM (*n* = 3). A schematic representation of the variant used in each system is shown above. (***p ≤ 0.001, **p ≤ 0.01, *p ≤ 0.05 vs. CaM, ANOVA was used).

We used a reporter with a 21‐residues‐long tether, previously found to correspond with a CaM‐dependent peak in the FPA (from now on referred to as the CRD L21 co‐translational folding biosensor; Urrutia et al., 2021). The results revealed a significant signal reduction of the *R*_FL_ for both CaM12 and CaM34 versus WT CaM (Figure 2a). However, folding was not totally abolished; the relative full‐length protein remained significantly higher in both mutants than in the absence of CaM, suggesting that while the ability of CaM to bind Ca^2+^ is beneficial in enabling folding of the CRD, it is not strictly required.

*In vitro* qualitatively similar results were obtained: the folding force of the CRD was decreased, but not completely abolished, for both CaM12 and CaM34 (Figure 2b).

In Figure 2, second column, we observe that in the absence of CaM there is a difference between the *in vivo* and *in vitro* conditions. This likely reflects the presence or absence of cellular quality‐control mechanisms, including ribosome rescue pathways (Kurita & Himeno, 2022). In bacteria (*in vivo*), misfolded or stalled CRD polypeptides may be prematurely released or degraded, resulting in almost undetectable expression without CaM. In contrast, *in vitro* translation systems, which contain only ribosomes and the minimal translational machinery, lack these rescue pathways, allowing more CRD to be synthesized even in the absence of CaM. This result also suggests that probably some early‐folding events take place in the CRD that generate enough pulling‐force to restart the synthesis and release full‐length proteins, increasing the *f*_FL_. However, the difference between the *in vitro* translation in the presence and absence of CaM is still significant, suggesting an inability of the CRD to fold properly without CaM.

### 2.3 hA has a preponderant role in folding of the CRD

**FIGURE 3:**
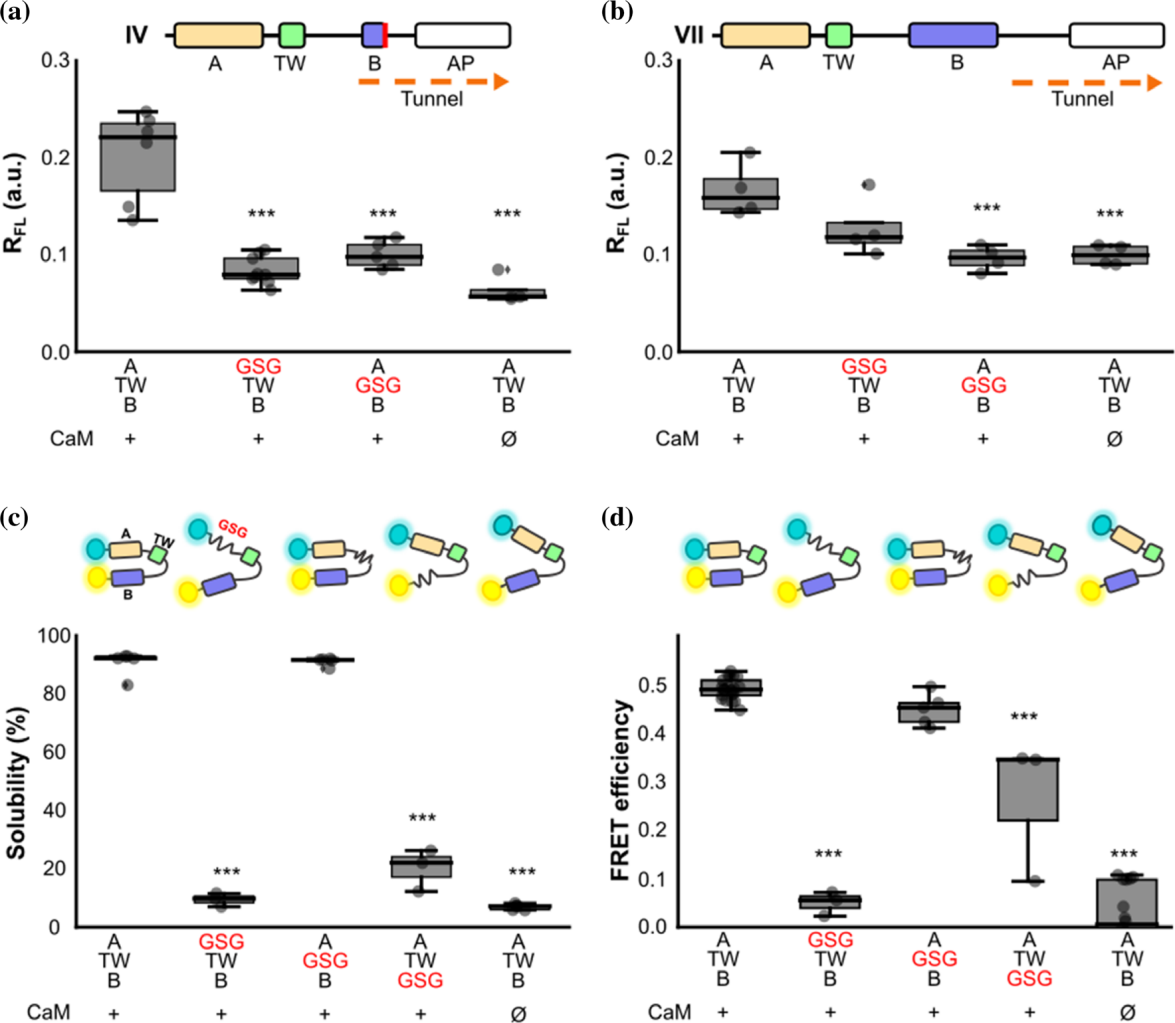
Impact of each of the CRD helices in FPA. (a) Box plot of the *R*_FL_ of the variant corresponding to peaks IV and (b) VII with a Gly‐Ser repeat sequence substitution in hA or in TW co‐expressed with CaM. (*n* = 4, except for *n*_IV(WT+CaM)_ = 6, *n*_IV(GSG in hA)_ = 9, *n*_IV(GSG in hTW)_ = 5). Schematic representation of the WT (A‐TW‐B) variant corresponding to IV and VII is indicated at the top. (c) Solubility: box plot of the percentage of the fluorescence intensity of the supernatant band of SDS‐PAGE gels. (*n* = 3, *n*_GSG for hTW_ = 5). (d) Box plot of FRET efficiency: Values obtained from spectra of the soluble fraction. (*n* = 3, 5, 3, respectively for hA‐, hTW‐ and hB‐disrupted‐CRD). In the upper part of both panels, a and b, there is a schematic representation of the biosensors. Note that the separation between hA and hB in the cartoon representation reflects low FRET values. (*n* = 3, except for *n*_GSG in hTW_ = 5). Asterisks refer to the significance compared to the WT variants (***p ≤ 0.001 and **p ≤ 0.01, ANOVA) for all the panels.

After establishing the overall impact of CaM on CRD folding, we aimed to elucidate the native pathway followed by the nascent CRD during translation. We focused on two key reporters corresponding to peaks IV and VII (Figure 1), associated with exit of hTW and hB from the ribosomal tunnel, respectively. For each construct, each α‐helical domain was disrupted with GSG stretches to prevent folding, as indicated (Figure 3). At stage IV, where hTW presumably is just outside the tunnel, we found that CaM‐mediated folding of hTW is strongly dependent on proper formation of hA: GSG disruption of hA caused a significant reduction in the full‐length protein production, even though CaM was also present (Figure 3a). Disruption of hTW itself also caused a comparable reduction in *R*_FL_. The folding transition observed at stage IV must therefore involve folding of both helices (in complex with CaM). At stage VII, we found that folding of hB is in turn highly dependent on proper formation of hTW: GSG disruption of hTW resulted in *R*_FL_ values indistinguishable from those obtained for the WT in the absence of CaM (Figure 3b). Given that we already established that hTW is itself dependent on hA, one would expect that hB folding is also (indirectly) dependent on hA. Indeed, *R*_FL_ measured on the hA‐disrupted VII construct was partially decreased.

The native CRD is known to form an antiparallel hairpin in the presence of CaM (Bernardo‐Seisdedos et al., 2018). By measuring protein solubility and Förster Resonance Energy Transfer (FRET) between the N‐ and C‐terminal fluorophores (Figure 3c,d, respectively), we can thereby follow the formation of such antiparallel hairpins *in vivo*. We used this concept to monitor structure formation of the CRD upon perturbation of each α‐helix (Figure 3c,d). Disruption of the small hTW had little effect on the native helical hairpin: the fluorescence ratio (or apparent FRET efficiency) was only marginally decreased, and solubility remained unchanged. This is consistent with existing structural models, where hTW does not make close contacts with CaM (Archer et al., 2019; Bernardo‐Seisdedos et al., 2018). As expected, no helical hairpin formation was observed when hA was perturbed—both FRET and solubility were completely abolished. However, the same was not true for hB perturbation: although both FRET and solubility measurements were significantly reduced, they remained above the baseline reference in absence of CaM. This suggests the formation of a metastable helical hairpin between hA and hTW in the presence of CaM: the partial fold would allow moderate solubility, while also ensuring a 3D conformation that confers some energy transfer.

### 2.4 CaM binding induces novel helical hairpin formations

**FIGURE 4:**
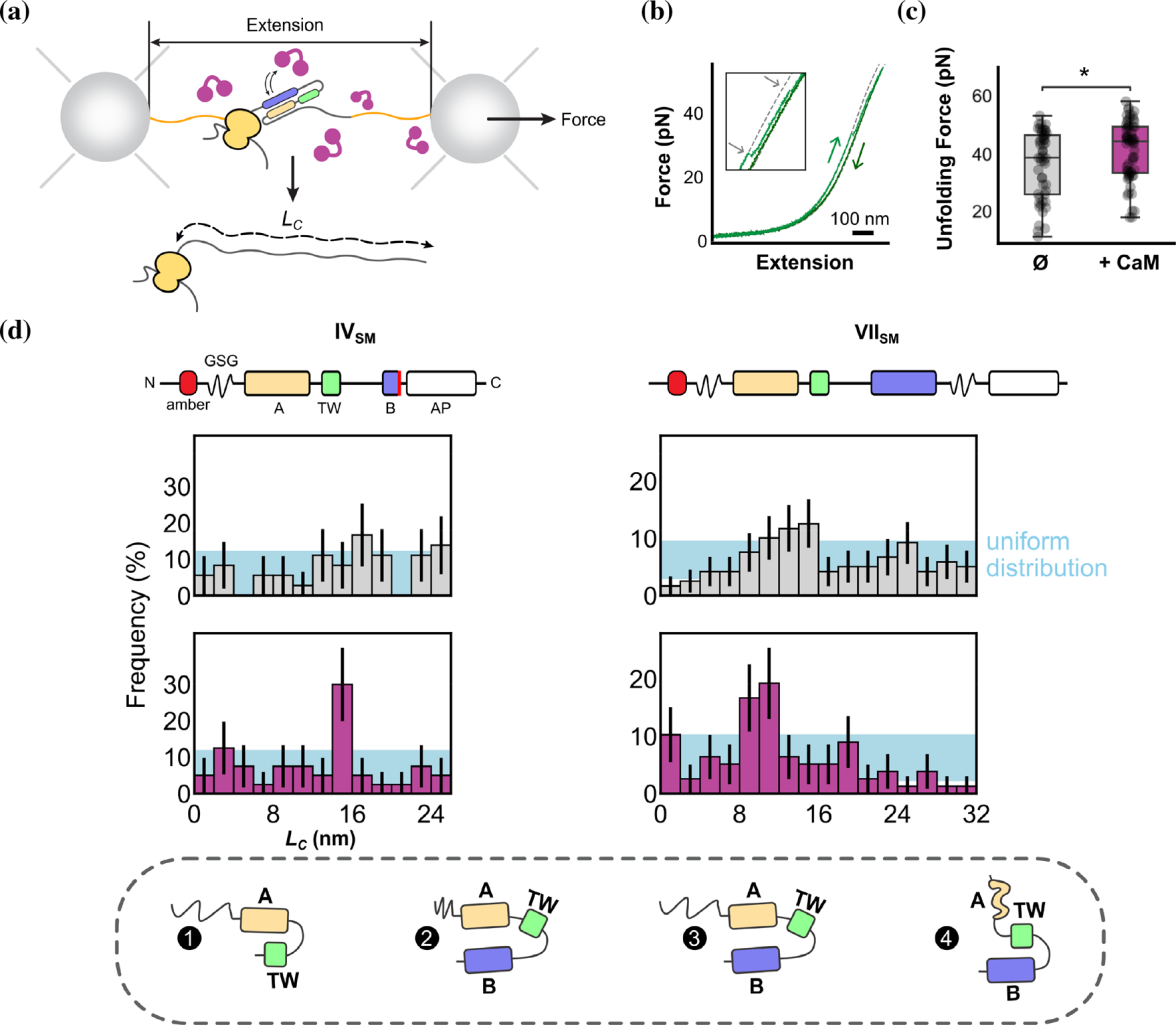
Identification of three CaM‐dependent unfolding states of nascent CRD. (a) Schematic representation of the experimental setup, showing a ribosome expressing the CRD (green) tethered between two optically‐trapped beads using DNA handles (orange). These experiments were performed both with and without added CaM (purple). (b) Example force‐extension data showing unfolding of the nascent CRD VII_SM_. In the experiment, the nascent chain is repetitively pulled (light green curve) and relaxed (dark green curve). From these force‐extension data, unfolding events can be identified (inset, gray arrows), and the unfolding force (*F*) and the end‐to‐end length of the unfolded part of the protein (*L*_*C*_) can be quantified. (c) Forces for all unfolding events of CRD VII_SM_ both with and without CaM. Binding of CaM to the nascent CRD stabilizes the domain against mechanical unfolding. (*p < 0.05). (d) Histograms showing all observed folding states, based on the *L*_*C*_ values measured from the force‐extension data, in the absence and presence of CaM (gray and purple, respectively) for the IV_SM_ and VII_SM_ variants (schematic representations of the two constructs are presented at top). Peaks are present at 14.9 nm for the IV_SM_ construct; and at 0, 10.2 and 17.7 nm for the VII_SM_ construct (Figure S5; Table S2). Bottom, schematic representation of the possible structures identified and described. States identified by force spectroscopy are consistent with the following structures: (1) a helical hairpin formed between hA and hTW (IV_SM_); (2) a fully compacted state with the entire CBD folded (VII_SM_); (3) the “native” state (VII_SM_) with the entire CRD folded and the N‐terminal tail fully extended; (4) a helical hairpin formed between hTW and hB, where the hA helix is unfolded and fully extended (VII_SM_). *N*‐values detailing number of experiments are given in Methods. For each construct, the distributions in the ± CaM conditions are significantly different (*p* = 3.2 × 10^−2^ and *p* = 8.1 × 10^−6^ for constructs IV_SM_ and VII_SM_, respectively; 2‐sample Kolmogorov–Smirnov test).

Next, we conducted an optical tweezers force spectroscopy assay to probe the conformation of single, nascent CRD molecules at nanometer resolution, both in the presence and absence of CaM (Figure 4a). We generated stalled ribosomes expressing CRD variants using *in vitro* transcription‐translation (Ohashi et al., 2010). Polystyrene beads were functionalized with stalled ribosomes using DNA “handle” linkers. After capturing each bead in a steerable optical trap, we changed the inter‐bead distance to repetitively stretch (Figure 4b, light green) and relax (Figure 4b, dark green) the nascent chain. Unfolding of the nascent protein during this stretching was marked by sharp discontinuities in the resulting force‐extension curve. Relaxation and waiting for 5 s. at 0 pN allowed the unfolded nascent chain to refold, while subsequent stretching at low forces was used to characterize the resulting folded state. Specifically, by fitting segments of the force‐extension data to the Worm‐like chain model, we quantified the contour lengths (*L*_*C*_) of the unfolded part of the chain. Hence, small *L*_*C*_ values representing more compacted states and large *L*_*C*_ values less compacted states. In addition, we characterized the forces at which these states unfolded (Figure 4b, gray arrows). This mechanical control allowed us to characterize the folding of the nascent CRD in the presence and absence of CaM, as a function of nascent chain length (i.e., IV and VII translation stages). Single‐molecule variants of these constructs (IV_SM_ and VII_SM_ respectively) were generated by inclusion of an amber codon and GSG sequence substitution, to enable tethering of the nascent chain.

The addition of CaM led to an increase in the average domain unfolding force (Figure 4c), which is consistent with mechanical stabilization of the nascent domain due to interactions with CaM. Note that this increase in unfolding force cannot be directly compared to measured free energy differences, as it reflects the stability of a range of conformational states (see below) and the unfolding reaction coordinates in mechanical pulling and temperature increase experiments differ.

In the absence of CaM, the conformation of the nascent CRD was highly variable, regardless of translation stage. At both stage IV and VII we observed a relatively uniform ensemble of states, ranging from fully‐compacted (*L*_*C*_ = 0) to fully‐unfolded (*L*_*C*_ = 28 or 34 nm; Figure 4d). The length distribution was statistically indistinguishable from uniform (blue bar; see Methods), indicating no statistical preference for a single protein conformation. The data did indicate, however, that nascent CRD can adopt folded states even in the absence of CaM, consistent with the FPA experiments (Figure 1d,e). When CaM was added, peaks were observed in the histogram at specific lengths. At the later translation stage VII, we found a peak at *L*_*C*_ ≈ 10 nm (Figure 4d, right). Using the known structure of the CaM/CRD complex (Bernardo‐Seisdedos et al., 2018), we could compute the expected contour lengths of various CRD conformations (see Methods). This analysis showed that *L*_*C*_ ≈ 10 nm is consistent with the native hA‐hB antiparallel hairpin conformation, where the intrinsically‐disordered N‐terminal domain (Bernardo‐Seisdedos et al., 2018) is extended by the applied force (Figure 4d, bottom). Further, it is interesting to note that CaM led to an increased population of states at *L*_*C*_ < 5 nm and *L*_*C*_ ≈ 18 nm, which are consistent with the same hA‐hB hairpin but a compacted N‐terminal domain, and with a potential hTW‐hB hairpin, respectively. Although the population of these states is below statistical significance, given the generic binding mode of CaM to alpha helices, it is thus possible that CaM also promotes other off‐pathway alpha‐helical hairpins.

At the earlier translation stage IV, one peak emerged in the presence of CaM with *L*_*C*_ ≈ 15 nm (Figure 4d, left). This length matches the expected contour length of the proposed hA‐hTW antiparallel hairpin, after accounting for the decreased nascent chain length. Notably, this state is only weakly populated in the VII distribution (Figure 4d, left column 1; *L*_*C*_ ≈ 25 nm, due to C‐terminal length differences) and is diminished, supporting the earlier observation that the presence of hB prevents CaM from forming the hA‐hTW intermediate.

**FIGURE 5:**
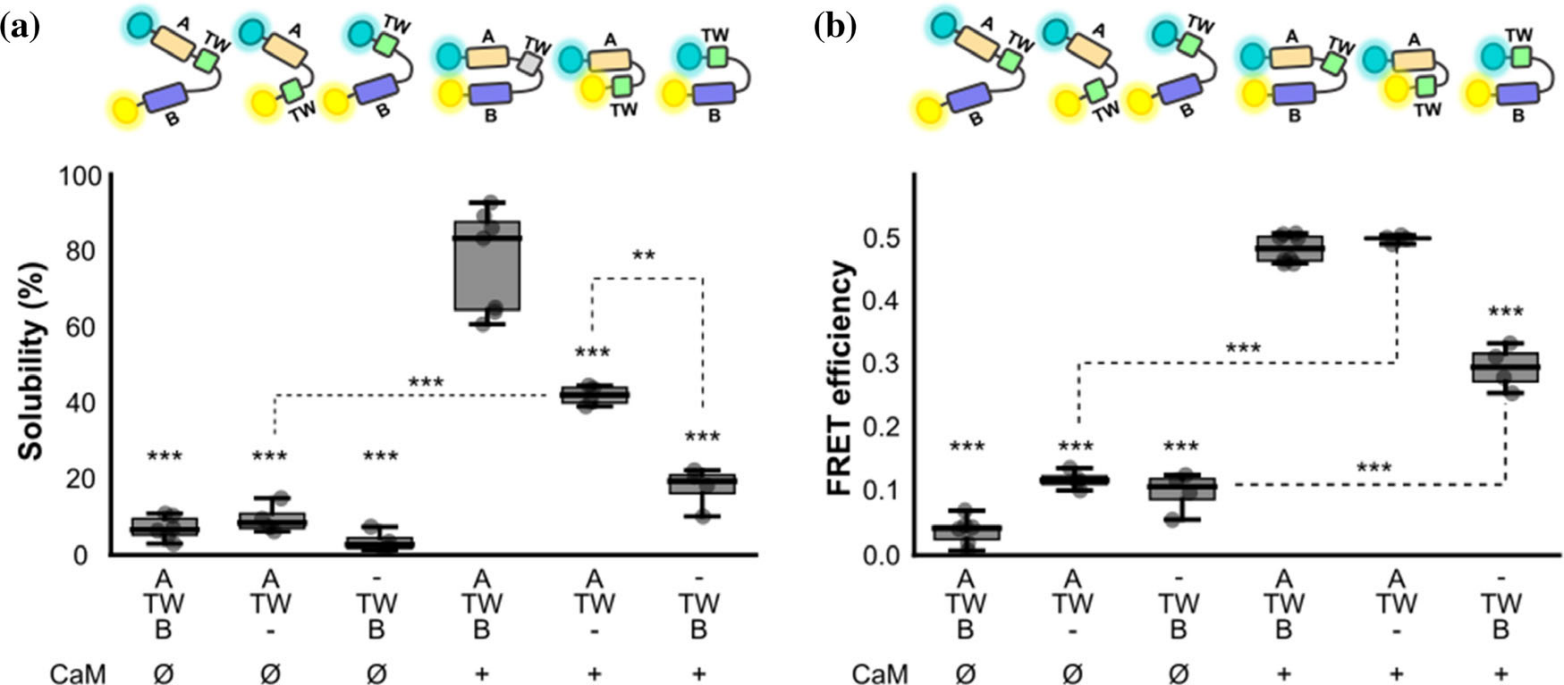
hTW forms a helical hairpin with hA in the presence of CaM. (a) Solubility: Fluorescence intensity of the supernatant band in SDS‐PAGE gels for the hA‐hTW‐hB, hA‐hTW and hTW‐hB biosensors without and with co‐expression of CaM. (*n* = 4, *n*_hA‐hTW‐hB+CaM_ = 7, *n*_hA‐hTW‐hB_ = 6). (b) FRET efficiency: Values obtained from spectra of the soluble fraction. (*n* = 4, *n*_hA‐hTW‐hB+CaM_ = 8, *n*_hA‐hTW‐hB_ = 6). Asterisks located at the top of each box refer to the significance compared to the WT biosensor in the presence of CaM (***p ≤ 0.001 and **p ≤ 0.01, ANOVA).

To obtain further insights, we modified the WT FRET reporter by eliminating hB or hA, leaving the hA‐hTW or hTW‐hB sequences flanked directly by the two fluorophores, respectively. Expression of the hA‐hTW construct in the presence of CaM resulted in a FRET index indistinguishable from that of the WT reporter (Figure 5b), with only moderately decreased solubility (Figure 5a), again indicating that CaM can indeed stabilize an antiparallel hairpin between hA and hTW alone. However, FRET and solubility were significantly reduced for the hTW‐hB construct in the presence of CaM, probably indicating that this hairpin is less stable and may represent a transient folding pathway (Figure 5).

In addition, as mentioned above, we employed AlphaFold Multimer to predict three‐dimensional conformations of these nascent chains at each arrested position (Evans et al., 2023; Jumper et al., 2021). The predicted structures were analyzed and selected based on consistency with our experimental data, revealing a plausible series of folding events for the nascent K_V_7.2 domain (Figure S6).

**FIGURE 6:**
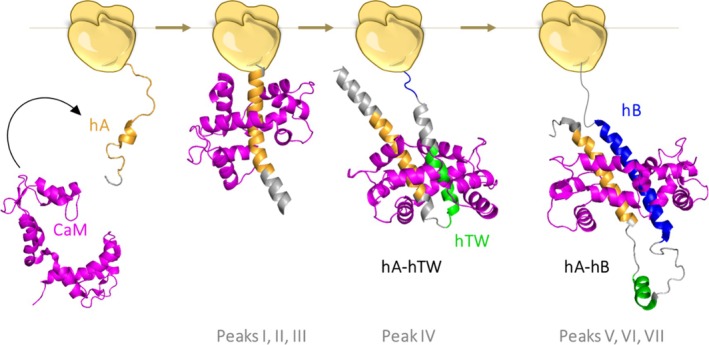
Schematic representation of a plausible co‐translational folding pathway. hA, hTW and hB are colored in orange, green and blue, respectively. CaM molecules are colored in magenta. Since the structure of hA‐K_V_7.2 in the absence of CaM is not available, the prefolded state of hA (left) was based on the prefolded state of the IQ‐like motif of SK2, (PDB: 1KKD). The middle states were obtained from running AlphaFold Multimer predictions of different sequences used for FPA and CaM. The final state (right) is that of the NMR structure of the K_V_7.2 CRD/CaM complex (PDB: 6FEH).

Figure 6 provides a schematic representation of this proposed co‐translational folding pathway. In this model, the prefolded state of helix A (hA) is shown in orange, helix TW (hTW) and helix B (hB) are colored in green and blue, respectively, while calmodulin (CaM) molecules are depicted in magenta. The middle states along the folding pathway were derived from AlphaFold Multimer predictions, corresponding to the sequences tested in our assays. These intermediates highlight critical stages of nascent chain folding, revealing the progressive structuring of the helices and the engagement of CaM as translation proceeds.

The final folded state illustrates the culmination of co‐translational folding, in which CaM is tightly bound to the fully formed helices, stabilizing the mature conformation of the K_V_7 domain. Overall, these data suggest a stepwise, co‐translational folding process, beginning with the initial formation of hA and culminating in the complete assembly of the K_V_7.2 CRD‐CaM complex.

## 3 DISCUSSION

In this study, we provide a biophysical analysis of how calmodulin (CaM) modulates the co‐translational folding of the Calcium Responsive Domain of Kv7.2 channels. Our findings reveal that CaM promotes productive folding of the nascent CRD chain by stabilizing intermediate conformations, thereby minimizing off‐pathway species and favoring the formation of the functional helical hairpin architecture.

### 3.1 CaM as a co‐translational folding assistant

Through force‐profile analysis (FPA), FRET measurements, and single‐molecule force spectroscopy, we demonstrate that CaM binding enhances the stability of the nascent CRD and increases the efficiency of ribosomal release (Figures 1d,e and 4c). These effects suggest that CaM interacts with emerging hydrophobic segments to mitigate premature collapse or nonproductive interactions with the ribosome surface (Kaiser et al., 2011). Importantly, this stabilizing effect is not static: CaM dynamically reshapes the folding landscape, such that nascent CRD is steered along a pathway involving the formation of hA‐hTW and hTW‐hB intermediates (Figures 4d and 6). This funneling effect indicates that its role extends beyond target recognition, to altering folding trajectories during synthesis.

Although FPA directly reports the fraction of full‐length product, this measurement acts as an indirect yet highly sensitive proxy for the pulling forces generated during translation. These forces may arise from nascent‐chain folding or from productive engagement with CaM, but FPA cannot discriminate between these contributions. As the method integrates all force‐generating events into a single observable, it does not reveal which specific step, such as folding of an individual helix, CaM binding, or/and subsequent conformational rearrangements, underlies the signal at a given translation stage. Nevertheless, the data indicate that CaM increases the frequency of stable native helical CRD structures as its helices emerge from the ribosomal tunnel.

The observed further indicates a co‐translational assembly process. Co‐translational assembly can occur between two nascent chains (Bertolini et al., 2021; Wruck et al., 2025) or between one nascent chain and a fully folded protein (Shiber et al., 2018). Here we show the latter for CRD and CaM. More broadly, protein steady‐state levels depend on the balance between synthesis and degradation, and many complex subunits are rapidly degraded or aggregated when they fail to associate with their cognate partners (Kamenova et al., 2019; Khan & Fox, 2023). An important aspect of co‐translational assembly is its capacity to buffer the deleterious effects of dominant‐negative mutations, which would otherwise allow a mutant allele to disrupt the function of the wild‐type counterpart (Natan et al., 2017).

Consistently, we have previously reported that, unlike productive CaM‐assisted folding, misfolded or co‐translationally trapped states, such as those described for the W344R mutant (Urrutia et al., 2021), appear unable to reach conformations that can be efficiently recognized by CaM *in vivo*, even though the isolated mutated CRD retains CaM‐binding competence after refolding *in vitro*.

Our data indicated that CaM exhibits chaperone‐like features, such as altering the folding energy landscape, promoting the presence of stable native states over alternative states, and improving substrate solubility. In addition, CaM is known to bind a diverse array of target proteins. We do not propose that CaM is a bona fide chaperone like GroEL‐ES (Naqvi et al., 2022), which can catalytically fold multiple protein substrates in sequence. Our data also do not distinguish whether CaM promotes stable structures by actively inducing CRD folded states (“induced fit” model) or more passively binds conformers that CRD adopts autonomously (“conformational selection” model), though we note that distinguishing these models is challenging in general, in particular for chaperones.

While our findings support a folding‐assistance function, whether CaM should be formally classified as a molecular chaperone remains a point of debate. Classical chaperones such as Hsp70 and CCT/TRiC are generalist ATP‐dependent machineries that assist a broad range of substrates. In contrast, CaM acts through specific and regulated target engagement. Nonetheless, the ability of CaM to stabilize folding intermediates, protect hydrophobic surfaces, and influence the timing of conformational transitions suggests that it performs *client‐specific chaperone‐like functions*. This concept aligns with prior observations that CaM can interact with and modulate components of the chaperone network, including Hsp70 (Huang et al., 2009) and Hsp40/Sis1 (Eisele‐Bürger et al., 2023). Thus, CaM may represent a specialized, signal‐regulated member of the broader chaperone *continuum*.

### 3.2 Integration with calcium signaling and cellular regulation

Our data further reveal that Ca^2+^ binding capability of CaM modulates its ability to stabilize CRD folding intermediates (Figure 2), suggesting that Ca^2+^ signaling may tune the folding assistance provided by CaM. This introduces a new layer of complexity to protein homeostasis: Ca^2+^ fluctuations not only regulate enzyme activity and trafficking but may also influence folding outcomes directly through CaM (Alaimo et al., 2009; Call & Hyson, 2016; Canclini et al., 2020; Chin et al., 1987; Etxeberria et al., 2008; Van Coppenolle et al., 2004). We propose that this dual role creates a functional link between signaling and proteostasis, allowing cellular conditions to dynamically modulate co‐translational folding efficiency.

It should be noted that Ca^2+^ binding was not directly manipulated or quantified in this study. Instead, we employed CaM mutants with impaired Ca^2+^ binding at defined EF‐hands as an experimental tool to evaluate Ca^2+^‐dependent effects. This strategy has been widely used to disentangle structural and functional contributions of Ca^2+^ binding to CaM‐target interactions (Geiser et al., 1991; Keen et al., 1999). While this approach does not allow direct assessment of Ca^2+^ occupancy during translation, it provides mechanistic insight into how Ca^2+^‐induced conformational states of CaM influence its folding‐assistance function.

### 3.3 Implications for channel biogenesis and disease

The coupling between CaM binding and folding may explain the observed requirement of CaM for ER exit of K_V_7.2 channels (Alaimo et al., 2009; Etxeberria et al., 2008). Since misfolded or partially folded proteins fail to traffic beyond the ER, the regulatory effects of CaM on ER export likely reflect its influence on co‐translational folding quality. Disease‐associated mutations that impair CaM binding (Urrutia et al., 2021) could thus disrupt not only the final CaM‐CRD complex but also the correct formation of intermediate states, such as the hA‐hTW and hTW‐hB hairpins. This mechanistic insight clarifies how these mutations produce trafficking defects and reduced channel currents in conditions such as benign familial neonatal epilepsy (BFNE) and epileptic encephalopathy.

Furthermore, previous reports indicate that CaM can modulate Sec61‐mediated translocation (Erdmann et al., 2011; Shao & Hegde, 2011), raising the possibility that CaM may coordinate membrane insertion and folding during translation. These functions may converge to ensure the fidelity of channel biogenesis.

### 3.4 Functional role of the hTW helix

Our results also shed light on the elusive function of hTW, a helix previously thought to be structurally dispensable (Gomis‐Perez et al., 2015). We demonstrate that although hTW is not essential for the final fold or for hA‐hB hairpin formation, it is crucial for co‐translational folding efficiency. The formation of sequential helical hairpins (hA‐hTW followed by hTW‐hB) acts as a folding relay that channels the nascent CRD toward the correct topology (Figures 3c,d, 4, and 5). Thus, hTW contributes kinetically rather than statically, explaining its disease relevance despite its minor impact on final channel conductance (Gomis‐Perez et al., 2015).

### 3.5 Model for CaM‐assisted co‐translational folding

By integrating our force spectroscopy, FRET, and computational modeling results (Evans et al., 2023; Jumper et al., 2021; Muguruza‐Montero et al., 2021), we propose a stepwise folding model in which CaM recognizes early helical precursors in hA as they emerge from the ribosome (Bhushan et al., 2010; Ramis et al., 2023; Ziv et al., 2005), stabilizes transient hairpins involving hTW, and finally promotes formation of the native hA‐hB helical hairpin once hB is fully synthesized (Figure 6). Whether CaM operates through conformational selection or induced fit remains an open question (Muguruza‐Montero et al., 2021). In any case, its presence reshapes the co‐translational folding landscape toward productive folding.

### 3.6 Limitations and future directions

A limitation of this work is the use of *E. coli*‐based *in vivo* force profiling, as direct co‐translational folding assays are not yet feasible in eukaryotic cells (Liutkute et al., 2020; Picking et al., 1992; Voss et al., 2006). While the bacterial system ensures CaM‐free baselines, eukaryotic ribosomes differ significantly in tunnel geometry and chaperone interactions. Developing eukaryotic‐compatible arrest peptides (Shanmuganathan et al., 2019; Yanagitani et al., 2011) and translation‐coupled force assays will be critical to extend these findings to native systems.

We also studied a truncated CRD construct that folds autonomously and binds CaM, but this excludes N‐terminal and transmembrane domains (>350 amino acids) that could provide additional stabilizing or regulatory interactions. The physiological relevance of CaM‐assisted folding in the full‐length channel thus warrants further testing under conditions mimicking the membrane insertion and ER environment.

### 3.7 Broader implications

Collectively, our findings suggest that CaM is capable of functioning as a co‐translational folding modulator, bridging signal transduction and protein biogenesis. This role, though distinct from canonical ATP‐dependent chaperones, represents a specialized regulatory form of chaperoning: one that is guided by signaling state, substrate specificity, and temporal coupling to translation. Given the diversity of CaM targets across the eukaryotic proteome (Alaimo & Villarroel, 2018; Urrutia et al., 2019), similar mechanisms may operate in other CaM‐dependent proteins, linking cellular signaling with folding fidelity and disease manifestation.

## 4 MATERIALS AND METHODS

### 4.1 Library of co‐translational folding biosensors

To perform the force profile analysis *in vivo*, 29 constructs were created by sequentially shortening the K_V_7.2 CRD (isoform 3, residues 316–545 with (ΔR373‐T501); Bernardo‐Seisdedos et al., 2018) residue by residue at regions of interest (Figure S1). We chose to use the Δ6L deletion because the wild‐type Kv7.2‐hAB fragment is insoluble and prone to aggregation. In contrast, the Δ6L construct produces a soluble, monodisperse complex with CaM and preserves the functional properties of the wild‐type channel fragment (Bernardo‐Seisdedos et al., 2018). CRD domains of variable length were followed by the arrest peptide (AP) SecM (*Ec‐Ms*) (FSTPVWISQHAPIRGSP) and flanked by mTurquoise2.1 (241 residues; 27 kDa) and mcpVenus (263 residues; 29 kDa) in the N‐ and C‐terminal, respectively. These constructs were cloned into the pProEx‐HTc by GenScript. Helices A or TW from CRD were then replaced by a Gly‐Ser repeat sequence of the same length as the helices in some of these constructs (corresponding to peaks IV and VII from the FPA; Figure 4; Figure S2).

These variants were cloned into the pET19b plasmid using Gibson Assembly for *in vitro* transcription/translation. Since the N‐ and C‐terminal of the designed constructs were identical, this technique enabled high throughput cloning using the same four primers for all inserts (see Supplemental Text S1 for further information about Gibson Assembly). In these clones the mTurquoise2.1 was removed, starting directly from the CRD, and the C‐terminal mcpVenus was replaced by a LepB P2 domain‐derived sequence encoding 23 residues (GSSDKQEGEWPTGLRLSRIGGIH) to enable radioactive quantification.

To analyze the relative full‐length protein ensuring that the entire CRD has exited from the ribosomal tunnel, an already described co‐translational folding biosensor was used (Urrutia et al., 2021). This consists of the CRD followed by a 21‐residue‐long linker including the 17 residues of the SecM (*Ec‐Ms*) (EFYV‐FSTPVWISQHAPIRGSP) (referred to as L21) coding sequence and flanked by mTFP1 and mcpVenus fluorescent proteins in the N‐ and C‐termini, respectively, and cloned into pProEX‐HTc plasmid (Figure S1). This variant was also cloned in pET19b plasmid for *in vitro* transcription/translation.

Note that all these co‐translational folding biosensors were used to calculate the fraction of full‐length which reports on folding during translation as the relative full‐length protein. However, to analyze the final folding of the protein other biosensors were used (folding biosensors) using solubility and FRET efficiency as measurements. The WT folding biosensor, which was already described (Bernardo‐Seisdedos et al., 2018), consists on the CRD flanked by mTFP1 and mcpVenus fluorescent proteins in the N‐ and C‐termini, respectively, and is cloned into the pProEX‐HTc plasmid. This biosensor reports on the folding of the helical hairpin between hA and hB, indicated as an increase in FRET efficiency. In addition, each helix of the CRD was independently replaced by a Gly‐Ser repeat sequence in separate folding biosensors (Figure S3).

### 4.2 Mutant calmodulin versions

CaM12, CaM34, and CaM1234 in pOKD4 vector were synthesized by GenScript Biotech Corporation (Netherlands). CaM12 has a mutation in the EF1 and EF2 (D22A and D58A, respectively), CaM34 in the EF3 and EF4 (D95A and D131A, respectively), and CaM1234 has mutations in all the EF‐hands (D22A, D58A, D95A, and D131A).

### 4.3 *In vivo* expression for force profile analysis (FPA)

The library of pProEX‐HTc plasmids coding increasing lengths of K_V_7.2 CRD constructs was transformed in *E. coli* BL21 DE3 cells, either alone or with the pOKD4 plasmid carrying the CaM coding gene. Cells were grown overnight at 37°C from single colonies, and then diluted into 10 mL of fresh LB (1:100 dilution) and incubated at 37°C until reaching an OD_600_ of 0.6. Protein expression was induced during 3 h at 37°C by the addition of 0.5 mM IPTG. Cells were harvested by centrifugation at 5478*g* (7000 rpm with Eppendorf F‐35‐6‐30 Rotor for 5430/5430R rotor) for 5 min. The cell pellets were resuspended in 500 μL lysis buffer (50 mM HEPES, pH 7.4, 120 mM KCl, 5 mM NaCl, 5 mM EGTA, 0.5 mM dithiothreitol (DTT), and protease inhibitors (1X Complete; Roche Applied Science), and 1 mM PMSF), and similar OD values were fitted for all the samples. The cellular cultures were sonicated using 3 cycles of 10 s on/10 s off, and centrifuged at 19,000×*g* during 30 min at 4°C for supernatant and pellet separation. The pellets were resuspended in the same buffer volume used before.

The soluble protein fractions were analyzed in a Fluoromax‐3 fluorometer by recording the emission spectra of mTurquoise or mTFP1 and mcpVenus fluorescent proteins upon excitation at 440 or 458 and 515 nm, respectively.

In this experimental setup, the synthesis outcome of these variants or folding biosensors is indicative of the pulling force exerted on the nascent chain and related to the folding status during translation. If the protein is stalled, an arrested protein (A) will be produced. In contrast, if folding of the partial CRD occurs during translation, the full‐length protein (FL) will be synthesized (Figure 1a). Thus, quantifying the stalling efficiency (*R*_FL_) provides a measure of co‐translational folding events. This is computed as the ratio between the emissions at the peak wavelength for Venus and for mTurq or mTFP1. This analysis is conducted after a 3‐h expression period, both in the presence and absence of CaM, within *E. coli*.

The relative full‐length protein (*R*_FL_) in the FPA was studied by SDS‐PAGE electrophoresis (15% acrylamide gels) using unboiled samples. The gels were visualized using Versadoc imaging equipment, exciting using blue or green LEDs, combined with 530BP28 or 605BP35 emission filters. After merging both images, the in‐gel *R*_FL_ was computed as the proportion of FL present in the soluble samples (*R*_FL_ = *I*_FL_ = *I*_FL_/(*I*_FL_ + *I*_A_)), by quantifying the fluorescent intensity of the bands corresponding to full‐length (*I*_FL_) and (*I*_A_) using FIJI (ImageJ) software.

### 4.4 *In vitro* expression for FPA

*In vitro* expression and analysis were conducted as previously described (Nilsson et al., 2017). Briefly, a linear DNA product was generated from each construct plasmid by PCR using Q5 polymerase with forward and reverse primers that anneal to the T7 promoter and terminator regions, respectively. Following PCR cleanup (using the manufacturer's instructions), the product was confirmed by agarose gel electrophoresis. *In vitro* transcription and translation were carried out in the PURExpress commercial system (mixed according to the manufacturer's recommendations). One hundred nanograms of the PCR product of K_V_7.2 CRD constructions were added into the reaction and *in vitro* co‐expression of CaM was carried out in a CRD:CaM 1:1 or 1:0.5 ratio adding the PCR product. PCR product(s) and ∼8 μCi of [^35^S]‐methionine were mixed for a 15‐μL PURExpress reaction, followed by incubation at 37°C for 30 min at 700 rpm shaking. Translation was ceased by the addition of 10 μL of ice‐cold 10% trichloroacetic acid (TCA), followed by incubation on ice for at least 30 min. Total protein was sedimented by centrifugation at 4°C for 5 min at 20,000*g*. The supernatant is carefully removed and the pellet was resuspended in a suitable volume of 1× SDS/PAGE sample buffer (134 mM Tris·HCl at pH 8, 13.5% (v/v) glycerol, 3.32% SDS, 0.075% bromophenol blue, 10 mM EDTA and 100 mM DTT) by shaking at 37°C and 900 rpm for 10 min. The prolyl‐tRNA that remains attached due to SecM arrest is digested by the addition of 4 μL of a 4 μg/μL RNase I solution, followed by incubation at 37°C and 700 rpm for 10 min.

Following a brief centrifugation (2 min at 20,000*g*) to remove any remaining insoluble material, the sample is loaded onto an appropriate SDS/PAGE gel (12% Bis‐Tris gels were used for large constructs and 16% Tricine gels were used for small constructs of the library run in MES or Tricine buffer, respectively). Following electrophoresis, the gels are dried onto thick filter paper by heating under vacuum (Bio‐Rad model 583 or Hoefer GD 2000), a radioactive molecular weight ladder included in the gel is visualized by spotting the filter paper with a ∼1:1000 solution of [^35^S]‐methionine in 1× SDS/PAGE sample buffer, and the gel is exposed to a phosphorimager screen for 12 h. The screen was imaged using a Fujifilm FLA9000 (50‐μm pixels), and densitometry analysis on the resultant raw image (TIFF format) file was carried out using FIJI (ImageJ) software. The densitometry values are quantified using our in‐house EasyQuant software and the fraction of full‐length protein was calculated. See Figure S4C,E for examples of gels. Independent replicate *in vitro* translation reactions were conducted for all K_V_7.2 CRD constructs.

### 4.5 Solubility and FRET assay

Protein expression and bacterial lysis were conducted as in FPA. The soluble protein fractions were analyzed in a Fluoromax‐3 fluorometer, recording the emission spectra of mTFP1 and mcpVenus fluorescent proteins upon excitation at 458 and 515 nm, respectively. FRET index was calculated as the ratio between the mcpVenus and mTFP1 emission peak amplitudes after exciting at 458 nm. FRET index was established as the ratio of emission at 520–525 divided by emission at 485–490 nm upon excitation at 456–460 nm. The total protein in each condition was assessed by direct excitation of the mcpVenus at 515 nm, collecting the emission at 520–570 nm. FRET efficiency was computed from the FRET index using the transfer function:

$$\text{Efficiency}=-0.9279+1.9335\times \left(\text{FRET index}/\left(\text{FRET index}+0.6821\right)\right).$$

The parameters were estimated by non‐linear fitting (*R* ^2^ > 0.99). The relationship between FRET index and FRET efficiency was computed using excitation and emission spectra for donor and acceptor, with a quantum yield of 0.85 for mTFP1 and 0.64 for mcpVenus, and *R*_0_ = 59.82 Å (www.fpbase.org/fret).

Protein solubility was analyzed by in‐gel densitometry, comparing protein amounts in equal volume of pellet and supernatant fractions. The protein amount in the pellet and in the supernatant was estimated relative to the total protein amount by quantifying the gel bands using FIJI (ImageJ) software. The gels were run as in FPA protocol.

### 4.6 Single‐molecule force spectroscopy assay

Detailed methods for single‐molecule sample preparation, optical tweezers assay and data analysis procedure are provided in the Supporting Information.

Briefly, variants that showed peaks IV and VII in the *in vivo* FPA (Figure S1) were adapted for single‐molecule assay through the incorporation of a N‐terminal amber codon with a flexible linker (RGSRGSGV), to enable the N‐terminus to be attached to the DNA handle and thereby allow mechanical manipulation. The SecM sequence was replaced by the stronger SecMstr variant (FSTPVWIWWWPRIRGPP) (Cymer et al., 2015; Kempf et al., 2017). dsDNA “handles” were prepared by PCR amplification with digoxygenin and biotin 5′‐end‐modified primers and purified on an agarose gel. The purified handles were incubated with Neutravidin to quench the exposed biotin tag.

Polystyrene beads were sequentially functionalized with dsDNA handles and biotinylated ribosomes by incubating them with the substrate for 20–60 min, followed by washing with TICO buffer (20 mM HEPES‐KOH, 10 mM (Ac)_2_Mg, 30 mM AcNH_4_ and 4 mM β‐mercaptoethanol at pH 7.4). Expression of the CRD by the bound ribosomes was induced using a ribosome‐free version of the PURExpress *in vitro* transcription‐translation kit, supplemented with the plasmid of interest and synthetic biotinylated lysine tRNAs. A second sample of beads was functionalized with dsDNA only using the same method.

Single‐molecule experiments were performed using a C‐Trap. Samples were measured in an environment containing a second “measurement” buffer (10 mM Tris–HCl, 250 mM NaCl, and 10 mM CaCl_2_ at pH 7.0), purified CaM (1 μM in the +CaM condition only), and an oxygen radical scavenging system (Swoboda et al., 2012). One RNC‐bead and one DNA‐bead were trapped. A single‐molecule tether was formed, then repeatedly stretched and relaxed until breakage. The resulting force‐extension curves were fitted with a twistable worm‐like chain model to extract the protein contour length values reported. Specific conformations of the nascent chains were proposed by comparison of the measured contour lengths with theoretical values computed using a structure of the CaM‐bound CRD previously reported. All data analysis was performed using custom scripts in Python.

### 4.7 Statistical analysis

Values are presented as box plot, indicating the median, lower and upper quartiles and minimum and maximum values. The differences between groups were evaluated using the unpaired Student's *t*‐test or ANOVA with Mann–Whitney post hoc on SigmaStat Statistic (SigmaPlot 11), where values of *p* < 0.05 were considered significant. The number of replicates in each experiment is indicated in the figures' legends. In all figures an asterisk, double asterisks, and triple asterisks indicate significance at *p* < 0.05, *p* < 0.01, and *p* < 0.001, respectively.

In the contour length distributions in Figure 4d, error bars are the standard error in a proportion, given by $\sigma =\sqrt{\frac{p\left(1-p\right)}{n}}$ (Avellaneda et al., 2020).

The blue highlight in each histogram shows the standard error region expected for a uniform distribution with the same number of observations. Error bars which overlap the blue region are therefore statistically indistinguishable from a uniform distribution (*p* < 0.05).

### 4.8 Supplementary material description

Supporting Information includes a detailed experimental description (15 pages), encompassing Supporting Text with methods, Figures S1–S7, Tables S1–S3, and SI References. Additionally, a SI_Dataset is provided, containing AlphaFold‐predicted structural models in PDB format.

## AUTHOR CONTRIBUTIONS

Arantza Muguruza‐Montero and Alvaro Villarroel conceived the study and participated in its design and coordination. Arantza Muguruza‐Montero, Sara M‐Alicante, Eider Nuñez, and Janire Urrutia carried out experiments and contributed to figure preparation and manuscript preparation. Ane Metola and Gunnar von Heijne contributed to the *in vitro* experiments. Jack R. Tait and Sander J. Tans conceived the force spectroscopy assay; Jack R. Tait, Vanda Sunderlíková, and Alexandros Katranidis generated samples for force spectroscopy assay; Jack R. Tait performed the force spectroscopy assay and associated data analysis and figure preparation. All authors read and approved the final manuscript.

## CONFLICT OF INTEREST STATEMENT

The authors declare no conflicts of interest.

## Supporting information


**Data S1.** Supporting Information.

## Data Availability

The data that support the findings of this study are available from the corresponding author upon reasonable request.
